# Wafer-scale synthesis of a morphologically controllable silicon ordered array as a platform and its SERS performance†

**DOI:** 10.1039/d3ra04797k

**Published:** 2023-11-16

**Authors:** Jizhe Song, Sujuan Feng, Haonan Shi, Daotong Han, Guangqiang Liu

**Affiliations:** a Qufu Normal University School of Physics and Physical Engineering, Shandong Prov Key Lab Laser Polarizat & Informat Qufu 273100 P. R. China fengsj@qfnu.edu.cn gqliu@qfnu.edu.cn

## Abstract

In this study, we fabricated four different structures using single crystal silicon wafers for surface-enhanced Raman spectroscopy (SERS) applications. For single crystal silicon, different crystal orientations exhibit different physical and chemical properties. In chemical etching, the etching speed of different crystal planes also exhibits significant differences. We first used reactive ion etching (RIE) to process the surface of the substrate, and subsequently used KOH anisotropic wet etching technology to modify the surface of silicon wafers with different crystal orientations and produced four different results. In the RIE stage in an O_2_ atmosphere, the (110) silicon wafer formed a hexagonal hole structure, and the (100) silicon wafer formed an inverted pyramid hole structure; however, in the RIE-treated substrates in O_2_ and SF_6_ atmosphere, the (110) silicon wafer formed a pyramid with a diamond-shaped base, and the (100) silicon wafer showed a columnar structure with a “straw hat” at the top. The formation mechanisms of these four structures were elucidated. We also performed structure-related SERS characterizations of the four different structures and compared their performance differences.

## Abstract 2

Development of a method to prepare four different three-dimensional structures in almost the same experimental process, with separate descriptions of the surface-constructing mechanisms, and structure-related SERS characterizations.

## Graphical abstract

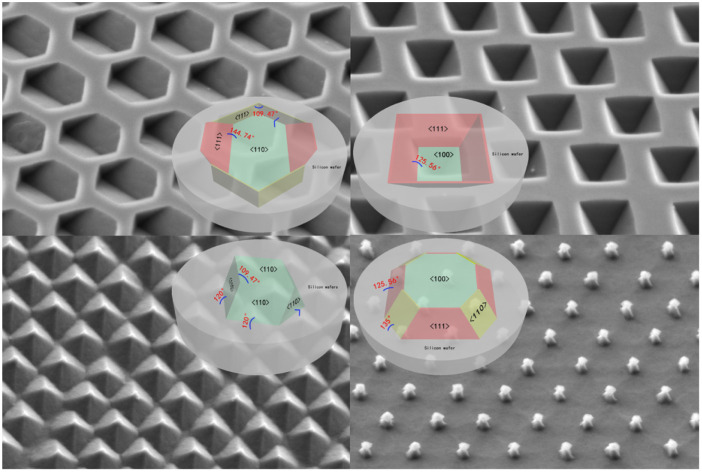

## 1. Introduction

Raman detection of molecules is an important means of determining their properties, but because of the extremely weak Raman signal, direct measurement is difficult to realize; therefore, researchers usually need to use SERS technology to amplify the Raman signal by several orders of magnitude. As SERS technology has received increasing attention, its commercialization is imperative. In addition to meeting the requirements of efficient, sensitive and easy to fabricate large-area regular and ordered arrays, the cost of commercialized SERS is also a factor that has to be considered. Monocrystalline silicon occupies an important position in the semiconductor field and is usually used as a SERS substrate owing to its superior optoelectronic properties, high stability, and low price. At present, in addition to sputtering deposition, some precious metals on the surface of silicon substrates,^1^ and surface structure modification of silicon is also a mainstream direction in SERS research to enhance the localized electromagnetic field of the enhanced surface, which can amplify the Raman signal by several orders of magnitude.^2,3^ Several methods have been reported for the surface structure modification of silicon, such as metal-assisted chemical etching (MACE),^4,5^ reactive ion dry etching,^6,7^ nanoimprint,^8^ chemical wet etching,^9,10^*etc.* The MACE method can not only guarantee the assembly of metal on the surface of the substrate but also has a single etching direction, which cannot form an ordered array structure. Although reactive ion dry etching can cooperate with the mask to form an anisotropic ordered array structure, the surface of the substrate also becomes rough under bombardment by a large number of high-energy particles inside the cavity. Similarly, nanoimprint technology also has extremely high requirements for the preparation process. Therefore, the development of a new method for the surface structure modification of silicon in SERS research is still of great significance.

In this study, we propose a new method, RIE, combined with wet etching. We were not only able to control the structure and morphology of the substrate surface by changing certain experimental conditions, but also took a step forward at the end of the RIE process and successfully obtained different types of substrate structures by anisotropic wet etching. We prepared four types of substrates and studied their etching mechanisms. SERS characterization confirmed their reliability in the detection of dye molecules. The preparation method is simple, inexpensive, and flexible, and substrates with different structures can be prepared according to different requirements. The reactions involved in the chemical wet etching used in this study are spontaneous chemical processes between molecules relying on crystal anisotropy; thus, the integrity of the substrate surface after etching can be ensured while the regular ordered array can be fabricated, which lays the foundation for the substrate to have a higher Raman signal intensity and signal escape rate.

## 2. Experimental method

Depending on the anisotropy of the crystal plane of the silicon wafer, various structures are formed during the wet etching.^11^ However, the biggest drawback of traditional wet etching is that it cannot manufacture regular and ordered arrays. In this study, regular ordered arrays were prepared using polystyrene microspheres (PS spheres) as masks during the reaction process.

**Fig. 1:**
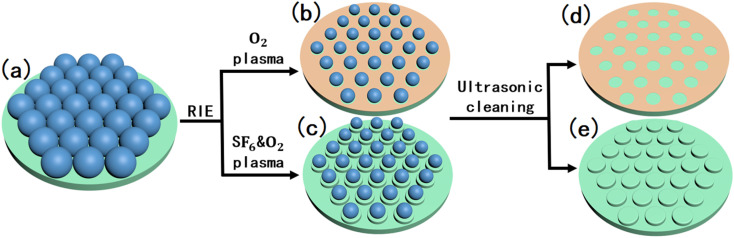
(a) Silicon wafer covered with a single layer of PS spheres; (b and c) PS spheres and silicon wafer after RIE etching; (d and e) the substrate was ultrasonically cleaned.

The silicon wafers were cleaned before the experiment. First, silicon wafers were ultrasonically cleaned with acetone, anhydrous ethanol, and deionized water for 10 min. Subsequently, the wafer was cleaned in a plasma cleaner for 180 s to improve the hydrophilic properties of the silicon wafer surface^12,13^ and then placed for later use. A certain amount of the PS suspension was mixed with absolute ethanol. After mixing, the mixture was ultrasonically cleaned in an ultrasonic cleaner for 2 min to eliminate PS pellet aggregates in the suspension. The silicon wafer was placed horizontally, an appropriate amount of deionized water was slowly poured onto its surface, and a pipette was used to inject the configured PS sphere suspension from the edge of the silicon wafer into the interface between the water surface and air at a constant speed. Under the action of the surface tension of the liquid, the PS spheres spontaneously formed regular and ordered hexagonal close-packed arrays at the liquid/gas interface.^14–16^ After being left to dry in natural shade at room temperature, a silicon wafer substrate covered with a monolayer of PS spheres was obtained, as shown in Fig. 1a. Fig. 1b and c show the RIE of a silicon wafer covered with PS balls under different gas conditions. The RIE conditions were divided into two types: one was only 20 sccm of O_2_, etching for 180 s; the other was the flow ratio of SF_6_ and O_2_ of 80 sccm : 100 sccm, and etching for 20 s. The power and high thresholds were 200 W and 50% respectively. In an O_2_ plasma environment, only the size of the PS spheres was reduced. Because the temperature of the reaction chamber table is as low as 11 °C, a layer of liquid water condenses on the surface of the chamber when gaseous water comes in contact with the etching table. After the radio frequency is turned on, the temperature in the chamber rises, and the liquid water turns into gaseous water molecules and mixes with the oxygen plasma. Although the surface of the silicon wafer is not etched, the reaction described in eqn (1) occurs in this atmosphere,^17–19^ oxide layer is generated in the unshielded regions of the PS spheres (shown Fig. 1b and d), which acts as a mask in the next KOH wet etching process.

1 Si + H_2_O → SiO_2_ + H_2_

In the O_2_ and SF_6_ plasma environment, in addition to the PS sphere size being reduced, the silicon wafers were also etched into cylindrical ordered arrays. Then, ultrasonic cleaning was performed with acetone, absolute ethanol, and deionized water for 5 minutes to remove PS spheres from the substrate, the shape of the substrate is shown in Fig. 1d and e. Finally, the substrate was dried and placed in KOH solution at 75 °C for wet etching. A detailed experimental flowchart is shown in Fig. S1.†

## 3. Results and discussion

### 3.1 Characterization

**Fig. 2:**
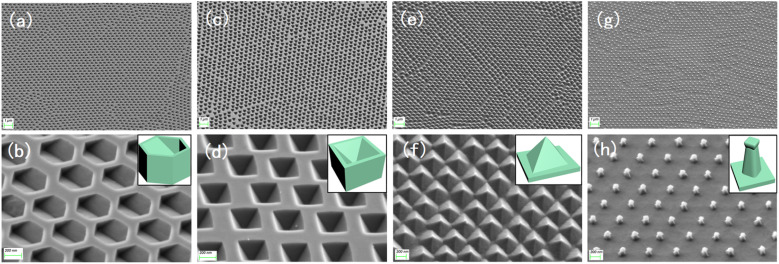
(a and b) Hexagonal array of (110) silicon substrate; (c and d) inverted pyramidal array of (100) silicon substrate; (e and f) diamond-shaped pyramidal array of (110) silicon substrate; (g and h) straw hat-shaped array of (100) silicon substrate.

Fig. S2b and c† show the different morphologies of the substrates under RIE under different atmospheric conditions. The structures shown in Fig. S2d and e† were obtained by cleaning the substrate in the previous step, and the residual PS spheres on both substrates were removed cleanly during the ultrasonic cleaning process. Fig. 2a and b shows the hexagonal hole array in the (110) silicon wafer obtained by wet etching for 40 s after O_2_ RIE, and the size of each hexagonal hole was approximately 350 nm. For the (100) silicon wafer, inverted tetragonal pyramid holes were obtained by wet etching for 40 s after RIE in the same atmosphere, and the size of each inverted tetragonal pyramid hole was approximately 300 nm, as shown in Fig. 2c and d. In O_2_ and SF_6_ plasma atmosphere, the final structures obtained were quite different from the above two results. As shown in Fig. 2e and f, for the (110) silicon wafer, the diamond-shaped pyramidal array structure is only etched by KOH for 20 s to form, and the (100) silicon wafer formed more faster, it only takes 15 s to appear a prismatic array with a “straw hat” shape on the top, which be shown in Fig. 2g and h.

### 3.2 Formation mechanism

As we all know, there are mainly three crystal planes in single crystal silicon, which are (110), (100) and (111) crystal planes, respectively. There are different atomic densities between different crystal planes, and there is a certain distance between the same crystal planes. These two factors together determine the physical and chemical properties of the crystal planes, so they will have corresponding rate differences when they are etched. For single crystal silicon, studies have shown that in an environment with only KOH solution, the (110) crystal plane has the highest etching rate, followed by the (100) crystal plane, and the (111) crystal plane has the slowest etching rate.^20,21^ Because of this anisotropic characteristic of single crystal silicon, in the wet etching process, the (110) and (100) crystal planes are always etched before the (111) crystal plane, and finally, some specific structures are formed.

**Fig. 3:**
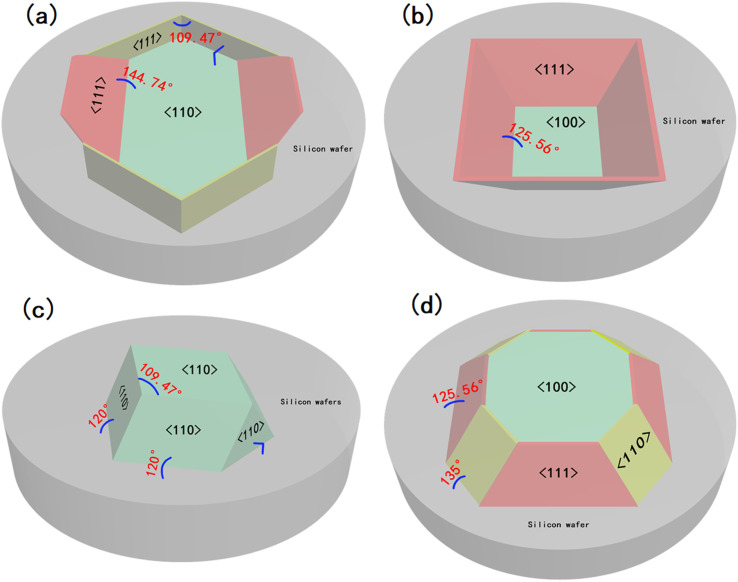
(a and b) Schematic diagrams of crystal planes of (110) and (100) silicon wafer substrates during wet etching after O_2_ RIE; (c and d) schematic diagrams of the crystal planes of the (110) and (100) silicon wafer substrates during the wet etching process after O_2_ and SF_6_ RIE.

First, after the initial preparation of the substrate by O_2_ RIE, the surface of the silicon wafer was kept intact; therefore, the crystal plane where the surface was located was first etched in the wet etching process. As shown in Fig. 3a, a total of six (111) faces are exposed during the etching process of the (110) silicon wafer, four of which are perpendicular to the horizontal plane, and the angle between the faces is 70.53°. The angle between the two obliquely intersecting crystal planes and the horizontal plane is 35.26°, while the other two (100) planes are located at both ends of the V-shaped groove at the bottom of the hexagonal structure, their angle with the (110) plane is 45°, which is not considered because it does not affect the formation of the structure. When the (110) face was etched downward, the six (111) crystal faces were exposed. As the (110) face is etched, the exposed area of the (111) face becomes increasingly larger, and eventually, the two inclined (111) faces intersect. At this time, only the (111) crystal plane is exposed, and the structure is completely formed. Continuing the etching will only change the size of the structure. Fig. 3b shows the etching mechanism of the silicon wafer with a (100) crystal orientation. In addition to the (100) plane, four inclined (111) planes are exposed, and the angle between them and the (100) plane is 54.44°. In addition, there are four inclined (110) planes located near the four edges of the inverted pyramid, and the angle between them and the (100) plane is 45°, because of the etching speed of the (110) plane is higher than that of the (100) plane, during the etching process only four (111) faces were displayed, so the (110) planes were not considered. Finally, the four (111) crystal planes intersected at one point at the bottom. At this time, the structure will not be changed by continuing to etch, and only the size of the structure will change.

**Fig. 4:**
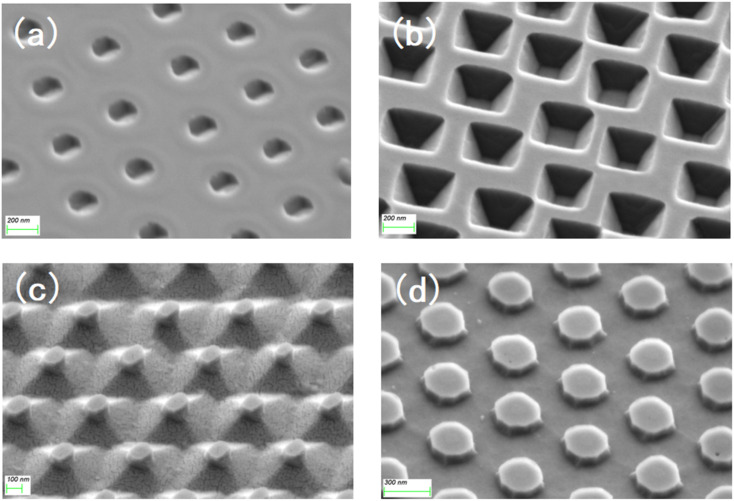
SEM images of (a) hexagonal hole structure, (b) inverted pyramid structure, (c) pyramid structure and (d) “straw hat” structure during etching process.

The substrates subjected to RIE in O_2_ and SF_6_ atmosphere were different from those in the O_2_ atmosphere. After etching was completed, the surface integrity of the silicon wafer was destroyed, and the whole was a cylindrical ordered array. Therefore, in addition to the surface, the crystal planes inside the crystal are also exposed on the side before wet etching, and all the exposed crystal planes are etched simultaneously. Fig. 3c shows the etching mechanism of the (110) Si wafer. During the etching process, six (110) planes were exposed in addition to the horizontal (110) plane, two of which were perpendicular to the horizontal plane, and the remaining four have an angle of 60° with the horizontal plane. While the horizontal (110) plane was being etched, the four inclined crystal planes quickly converged towards the middle, finally forming a pyramid structure with a rhombus-shaped bottom surface. For the (100) silicon wafer, some crystal planes were also exposed in advance for structural reasons, and a total of eight crystal planes were exposed in addition to the (100) plane during the etching process; the angle between the four inclined (111) faces and the (100) face is 54.44°, and the angle between the four inclined (110) faces and the (100) face is 45°. Since the etching speed of the (110) plane is much higher than that of the (111) plane, the (111) plane disappears when the four (110) planes intersect each other, because of the (110) surface is etched diagonally downward, a “straw hat” structure will be formed on the top. If etching is continued at this time, the tops of the four (110) planes meet at one point. At this time, almost no 3D structure was observed on the substrate. A schematic of the crystal plane is shown in Fig. 3d. The SEM images of the four structures at the mid-etching stage are shown in Fig. 4. Fig. 4a shows the image of the hexagonal hole-like structure during etching, and the inverted pyramidal structure shown in Fig. 4b does not have a transitional shape during etching. In Fig. 4c, although the previous RIE process was only 20 s, it still caused a certain amount of roughness on the side of the cylinder compared to the upper surface where the PS ball was shaded, which resulted in small differences in etching speed, making the side etched slightly faster. As the (110) plane was etched extremely fast, these slight differences were magnified and eventually a column was formed above the structure during the etching process due to the lower etching speed on the upper surface, but this column will eventually disappear as the etching process proceeds to the end. Fig. 4d shows an image of the etching process of the “straw hat” structure, in which the octagonal prism is formed first, and the “straw hat” structure is formed in the final stage of etching.

### 3.3 Structure-related SERS characterization

With the rapid development of the semiconductor industry in recent years, traditional monocrystalline silicon wafers have occupied a very important position in both photovoltaic power generation and integrated circuits. Owing to its superior optoelectronic properties, it is commonly used in the field of sensors. However, its defects are also obvious: when light irradiates the surface of the silicon wafer, only one reflection occurs, which results in only one photoelectric conversion between the photon and the substrate, as shown in Fig. S3a.† One of the mainstream methods for enhancing the efficiency of photovoltaic power generation is to fabricate ultra-black silicon, such as nanowires or nanopillar structures, which can trap most of the light on the surface of the substrate, thereby achieving high-efficiency energy utilization. Although ultra-black silicon has superior photoelectric conversion efficiency, its shortcoming is also obvious: because of the ultra-high absorbance and the intricate surface structure of the substrate, almost no optical signal can escape from it.^22^ Although the SERS mechanism relies heavily on the high photovoltaic conversion efficiency resulting from the Localized Surface Plasmon Resonance (LSPR) effect, it also requires a certain amount of signal to escape from the substrate. Therefore, ultra-black silicon is destined to be unable to be used in sensor fields such as molecular detection. Fig. S3b and c† show the reflection light path diagram of the surface of the hexagonal-structured substrate prepared by KOH wet etching. In most cases, light can be reflected twice or more on the surface, and in some cases, it can even be reflected on the surface with more than three reflections, which can prompt photons to interact with the substrate multiple times.

**Fig. 5:**
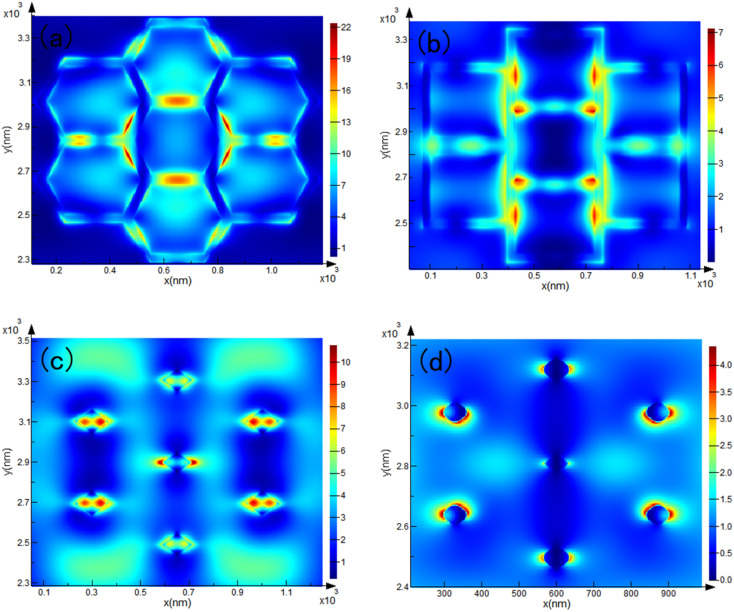
FDTD electric field enhancement simulation results for (a) hexagonal, (b) inverted pyramid, (c) pyramid, (d) straw hat prism substrates under 532 nm laser.

The Finite-Difference Time-Domain (FDTD) electric field-enhanced simulation results of the substrates with the four structures under excitation light at 532 nm are shown in Fig. 5. The monitors of the hexagonal and inverted pyramid structures are directly above the substrate, and put those at the lower third of the top of the pyramid and “straw hat” structures. The results show that the electromagnetic hot spot distribution of the modified substrate is uniform; therefore, the substrates have good electric field enhancement performance, which can significantly enhance the LSPR effect. Compared with other ultra-low reflectivity substrates such as silicon nanowires, we overcome the disadvantage that the Raman signal is trapped in the substrate and cannot escape during molecular trace detection. The photoelectric conversion efficiency was improved while maintaining a strong Raman signal escape rate, which can be used for the trace detection of molecules.

**Fig. 6:**
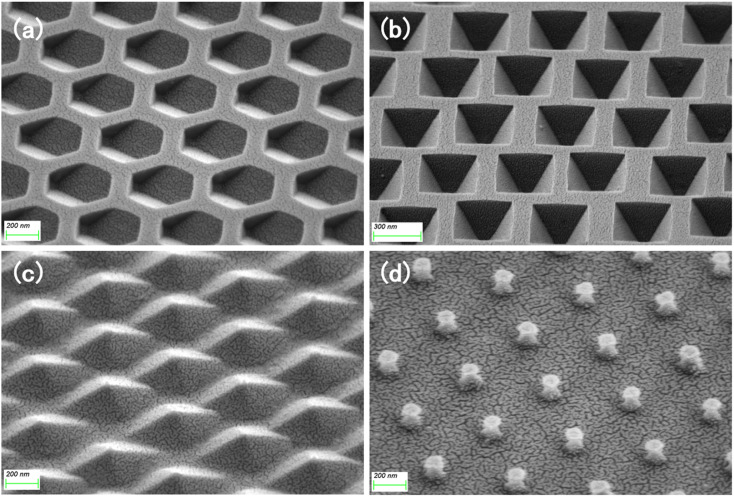
SEM images of (a) hexagonal hole, (b) inverted pyramid, (c) pyramid, and (d) “straw hat” structure after sputtering an Au film.

**Fig. 7:**
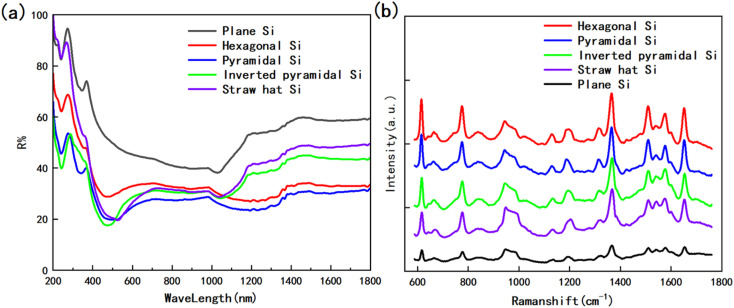
(a) Reflectance spectra of four structures of substrate and planar silicon in the range of 200 nm to 1800 nm; (b) Raman spectra of 10^−6^ M R6G on four substrates and planar silicon.

**Fig. 8:**
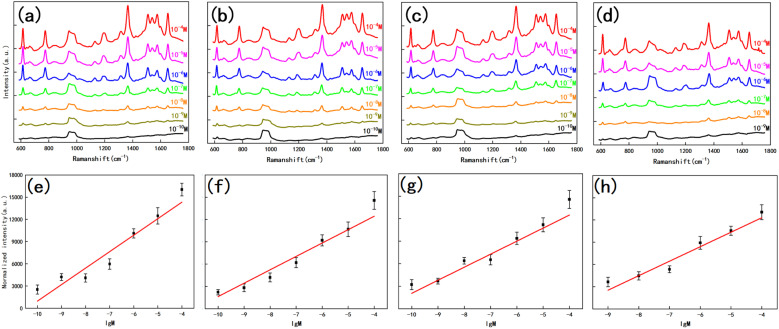
Detection limits of R6G for hexagonal (a), pyramidal (b), inverted pyramidal (c) and straw hat (d) shaped substrates; (e–h) correlation between the normalized intensity and concentration of the highest intensity eigen peaks in the Raman spectra of the above four structures.

To further enhance the contribution of the LSPR effect to SERS, both the substrate for the four structures and the planar silicon wafer were treated with magnetron sputtering gold for 20 s to ensure that the surface was uniformly covered with a layer of the Au film, as in Fig. 6. We measured the reflectance spectrum of the substrate in the range of 200–1800 nm, and the results are shown in Fig. 7a. After retouching the substrate, all four substrates had obviously lower reflectivity compared to planar Si, and a significant absorption peak appeared around 500 nm; therefore, in the next step of characterization of the Raman signal, we use a 532 nm laser for detection. After the slices were immersed in the prepared rhodamine 6G (R6G) solution (10^−6^ M) for 1 h, they were removed out and subjected to SERS signal detection. Fig. 7b shows the Raman spectra of the five substrates, and the substrates modified by wet etching showed a significant signal enhancement performance compared to the planar silicon wafer. In order to evaluate the sensitivity of the substrate, we used it to test the Raman signal of R6G solution with different concentrations respectively, as shown in Fig. 8a–d, as the sample concentration decreased, the signal intensity also decreased. The final hexagonal, pyramidal and inverted pyramidal structures can reach detection limits of 10^−10^ M. Even with the “straw hat” structure substrate the detection limit can still reach 10^−9^ M. And the substrates are sensitive to changes in the concentration of the detected substance, and there is a good linear relationship between Raman peak intensity and concentration, which can be used for quantitative analysis of target molecules. The fitted image of the correlation between the normalized intensity of the highest characteristic peak of the spectrum and concentration is shown in Fig. 8e–h.

**Fig. 9:**
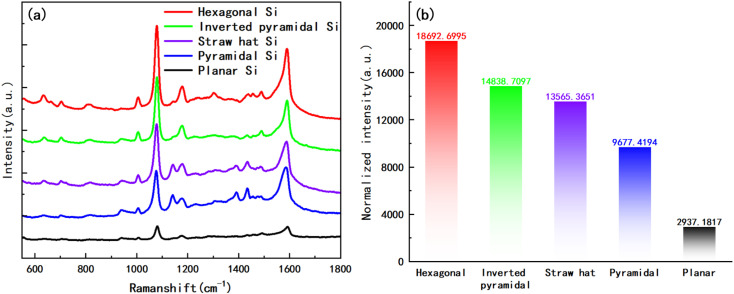
(a) Raman spectra measured after preparation of SAM with 4-ATP (10^−2^ M) on four structures and planar Si surfaces; (b) normalized intensities of the highest peaks of the Raman spectra of the five substrates in (a).

In addition, we also prepared self-assembled monolayers (SAM) of 4-Aminothiophenol (4-ATP) on their surfaces for the above four substrates and planar substrate. Firstly, we gold-plated the substrates for 20 s and then immersed them in a 4-ATP ethanol solution with a concentration of 10^−2^ M for 12 h. After taking out the substrates, they were respectively rinsed with anhydrous ethanol for 1 min continuously, in order to remove excess molecules on the surfaces and dried them in a vacuum drying oven. The Raman spectra measured with a 785 nm laser is shown in Fig. 9a, which indicate that the hexagonal structure of the substrate has the best SERS performance, followed by the inverted pyramid, and finally the “straw hat” and pyramid structures, all four structures have significant SERS enhancement in comparison with the planar substrate. It should be pointed out that four different substrates and the plane silicon surface are coated with gold film at the same condition (sputtered Au layer for 20 s), and they have similar nano-islands morphology, as shown in Fig. 6 and Fig. S4.† So, we think almost the same number of 4-ATP molecules adsorb on the surface of the Au nano-island structure within a unit area. At this condition, we compared the SERS performance of the four substrates and the plane silicon surface. Fig. 9b demonstrates the normalized intensity of the highest peaks in the Raman spectrum, where the hexagonal substrate has about 6.4 times the normalized intensity of the planar substrate, and even the pyramidal structure can reach 3.3 times.

**Fig. 10:**
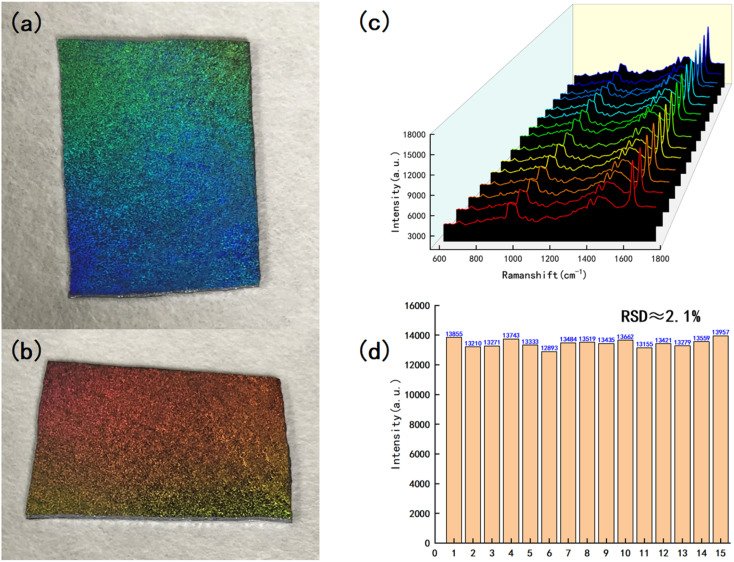
(a and b) Pictures of the prepared hexagonal hole structure of the substrate taken at different angles under natural light; (c) Raman spectra of MB (10^−4^ M) at 15 randomly selected points on the hexagonal substrate surface; (d) relative standard deviation of the intensity of the highest Raman peak in the Raman spectrum.

Reproducibility is an important factor for the quantitative analysis of target molecules in actual applications. Optical photographs of the substrate with a hexagonal hole structure are shown in Fig. 10a and b. The substrate has wonderful structured light properties, viewed from different angles in natural light, will show different colors, and looks like the wings of a butterfly. Using this method, we can synthesize four different structures on a 4-inch substrate, such as hexagonal hole substrate, and the optical photo was shown in Fig. S5.† To further prove its uniformity, we randomly selected 15 points on the hexagonal substrate surface for Raman detection of the methylene blue solution (10^−4^ M), as shown in Fig. 10c and d. The Raman spectra measured at 15 points showed no significant difference in the peak position or signal intensity. The relative standard deviation of the highest peak signal intensity is approximately 2.1%, which shows that the substrate has reliable repeatability and can be used for the trace detection of dye molecules.

## 4. Conclusion

In summary, we developed a new method-RIE combined with wet etching, to modify the surface structure of Si. Four different structures: hexagonal hole, inverted pyramid hole, pyramid, and columnar structures, were fabricated by controlling the experimental conditions, including the crystallographic orientation of silicon and atmospheres in the RIE stage in an O_2_ plasma atmosphere or in a mixture of O_2_ and SF_6_ Plasma atmosphere. The formation mechanisms of these four structures are proposed according to the principle of different crystal faces with different etching rates in the wet-etching process. Finally, we carried out SERS-related research on the substrates and proved that the substrates have good SERS performance in both uniformity and sensitivity and can be used for the trace detection of target molecules.

## Conflicts of interest

There are no conflicts to declare.

## Supplementary Material

RA-013-D3RA04797K-s001
