# Supplementary material for: Wafer-scale synthesis of a morphologically controllable silicon ordered array as a platform and its SERS performance
Source: RSC Adv. 2023 Nov 16;13(48):33625–33. doi: 10.1039/d3ra04797k (PMC10652251; doi:10.1039/d3ra04797k)
Supplement: RA-013-D3RA04797K-s001 [file RA-013-D3RA04797K-s001.pdf]

Electronic Supplementary Material (ESI) for RSC Advances.
This journal is © The Royal Society of Chemistry 2023

Supporting Information

Wafer-Scale Synthesis morphologically controllable silicon ordered array as platform and its SERS performance

Jizhe Song, Sujuan Feng, Haonan Shi, Daotong, Han, Guangqiang Liu**

Qufu Normal University School of Physics and Physical Engineering, Shandong Prov Key Lab Laser Polarizat & Informat, Qufu 273100, Peoples R China

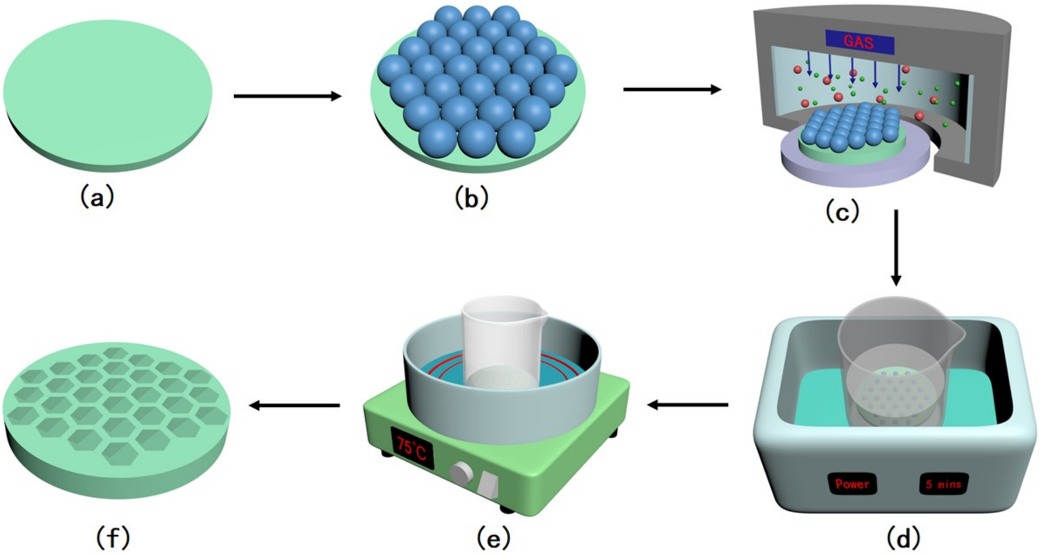

Figure S1. (a) Ultrasonic cleaning of silicon wafers with acetone, absolute ethanol, and deionized water for 10 min, respectively; (b) Single-layer PS sphere arrays were assembled on the surface of silicon wafers by liquid/gas interface self-assembly technology; (c) The silicon wafer with PS ball was subjected to RIE; (d) The substrate was ultrasonically cleaned with acetone, absolute ethanol, and deionized water for 5 min, respectively; (e) The substrate was wet-etched with KOH at 75 °C after drying; (f) Etching completed.

* To whom all correspondence should be addressed
E-mail: fengsj@qfnu.ecu.cn; gqliu@qfnu.edu.cn

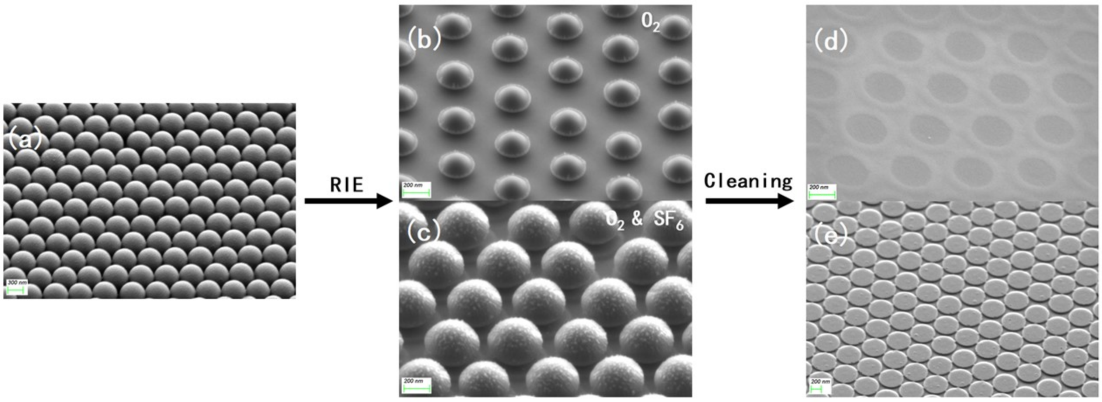

Figure S2. SEM image of (a) single-layer 500 nm PS microspheres prepared on a silicon wafer using liquid-gas interface self-assembly technology; (b) Substrate after O_2 RIE; (c) Substrate after O_2&SF_6 RIE; (d) Substrate ultrasonically cleaned after O_2 RIE; (e) Substrate ultrasonically cleaned after O_2&SF_6 RIE.

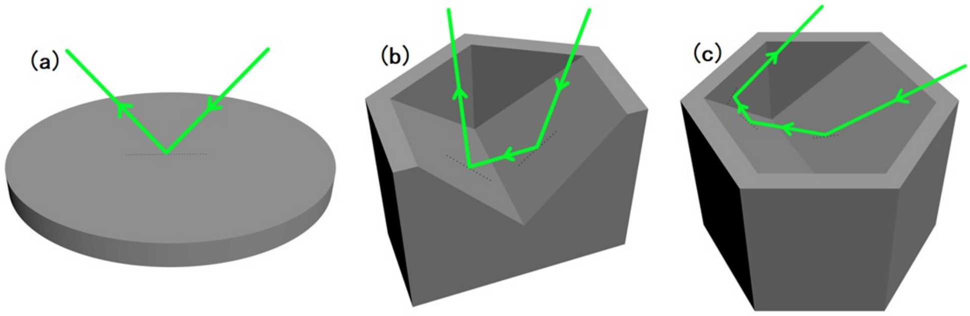

Figure S3. (a, b, c) Schematic diagrams of reflected light paths on different substrates.

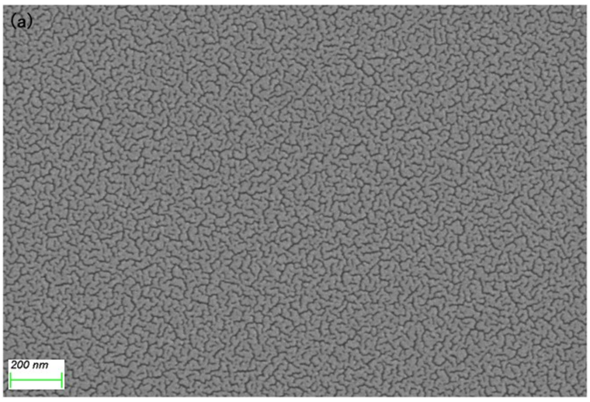

Figure S4. (a) SEM image taken after 20 s of gold plating on planar substrate.

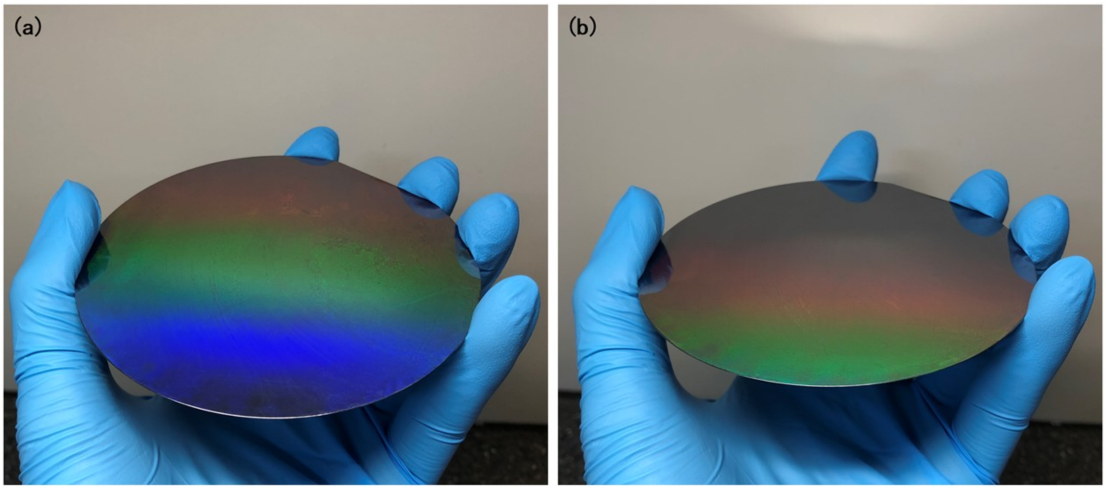

Figure S5. (a, b) Wafer-scale sized hexagonal hole substrate photographed at different angles.
